# Evolution of foot-and-mouth disease virus intra-sample sequence diversity during serial transmission in bovine hosts

**DOI:** 10.1186/1297-9716-44-12

**Published:** 2013-03-01

**Authors:** Marco J Morelli, Caroline F Wright, Nick J Knowles, Nicholas Juleff, David J Paton, Donald P King, Daniel T Haydon

**Affiliations:** 1 Institute of Biodiversity, Animal Health and Comparative Medicine, College of Medical, Veterinary and Life Sciences, University of Glasgow, Glasgow, G12 8QQ, UK; 2 The Pirbright Institute, Ash Road, Pirbright, GU24 0NF, UK; 3 Current address: Center for Genomic Science of IIT@SEMM, Istituto Italiano di Tecnologia at the IFOM-IEO Campus, Via Adamello 16, Milano, 20139, Italy

## Abstract

RNA virus populations within samples are highly heterogeneous, containing a large number of minority sequence variants which can potentially be transmitted to other susceptible hosts. Consequently, consensus genome sequences provide an incomplete picture of the within- and between-host viral evolutionary dynamics during transmission. Foot-and-mouth disease virus (FMDV) is an RNA virus that can spread from primary sites of replication, via the systemic circulation, to found distinct sites of local infection at epithelial surfaces. Viral evolution in these different tissues occurs independently, each of them potentially providing a source of virus to seed subsequent transmission events. This study employed the Illumina Genome Analyzer platform to sequence 18 FMDV samples collected from a chain of sequentially infected cattle. These data generated snap-shots of the evolving viral population structures within different animals and tissues. Analyses of the mutation spectra revealed polymorphisms at frequencies >0.5% at between 21 and 146 sites across the genome for these samples, while 13 sites acquired mutations in excess of consensus frequency (50%). Analysis of polymorphism frequency revealed that a number of minority variants were transmitted during host-to-host infection events, while the size of the intra-host founder populations appeared to be smaller. These data indicate that viral population complexity is influenced by small intra-host bottlenecks and relatively large inter-host bottlenecks. The dynamics of minority variants are consistent with the actions of genetic drift rather than strong selection. These results provide novel insights into the evolution of FMDV that can be applied to reconstruct both intra- and inter-host transmission routes.

## Introduction

Foot-and-mouth disease virus (FMDV) is a positive sense RNA virus, belonging to the *Picornaviridae* family, and is the causative agent of the highly contagious foot-and-mouth disease (FMD). RNA viruses evolve rapidly due to their large population size, high replication rate and poor proof-reading ability of their RNA-dependent RNA polymerase (quoted mutation rates commonly fall in the range of 10^-3^ – 10^-5^ per nucleotide (nt) per transcription cycle [1]). Within their hosts these viruses exist as complex, heterogeneous populations, comprising non-identical genome sequences [2,3]. Much of the genetic variation within FMDV populations is thought to be subject to neutral selection or to be under varying levels of purifying selection, with evidence for positive selection only observed in a small fraction of codons in the capsid and in non-structural proteins [4,5]. To facilitate rapid replication and intra-host dissemination, FMDV has evolved specific mechanisms to evade the early innate and adaptive immune responses, as reviewed in [6]. Infected hosts typically show clinical signs of FMD within 2–6 days post exposure that include vesicles on the coronary bands of the feet, in the mouth and on the tongue and teats [7]. Although alternative primary sites of replication have been studied (for a review, see [8]) rapid dissemination of FMDV from host entry most likely follows initial replication in the pharyngeal area [9-11]. Virus subsequently passes into the systemic circulation and is transported to other distant, non-contiguous epithelia, including those of the feet, where the virus can once again replicate. As a consequence of transportation of limited numbers of viruses to discrete replication sites these new local foci are founded by viruses that are likely to have passed through a population “bottleneck”, in the same way that virus populations are transmitted between hosts. The founder effects caused by these bottlenecks as the virus disseminates from the host inoculation site have been observed by conventional sequencing during serial FMDV infection in pigs [12] and by use of cDNA clones in poliovirus infection in mice [13].

An integral part of any disease control strategy is the epidemiological tracing of virus transmission, which, together with conventional field investigations, has largely been achieved with the application of molecular and phylogenetic methods [14-19]. Global tracing of FMDV movements have been successfully achieved using consensus sequences of the region encoding one of the three surface exposed capsid proteins of the virus (VP1) [16-18]. However, at shorter “epidemic” time scales, where the viral populations have not substantially diverged, VP1 sequencing cannot provide the required resolution. At this scale, complete genome consensus sequencing (CGCS) has proven to be a very powerful tool for transmission tracing [14,15,19]. Both the heterogeneous nature of within host viral populations and the number of transmitted viruses between hosts may influence the rate of fixation of mutations [20,21]; by only identifying the major viral sequence within a sample, CGCS masks the complex substructure of minority variants present and is therefore blind to subtle genetic differences between isolates that are closely related in space and time. Therefore, the level of resolution afforded by CGCS is inadequate to fully characterize single host-to-host transmissions and in particular to monitor the dynamics by which mutations accumulate over single transmission events. As a consequence, the processes that generate sequence variability at the intra-host scale that is transmitted on to the inter-host scale are still poorly understood.

Next-Generation Sequencing (NGS) techniques provide the means for rapid, cost-effective dissection of viral population dynamics at an unprecedented level of detail [22-29]. The resolution and high-throughput nature of NGS platforms has the potential to allow differentiation between samples at the inter- and intra-host scale of infection. This technology has already been applied to compare “longitudinal” samples of hepatitis C virus (HCV) and to study human immunodeficiency virus (HIV) infection and transmission [30-32]. These studies highlight the size of the population bottleneck during inter-host transmission as a likely influence on the long-term rate of nt fixation. In contrast to both HIV and HCV, where typically only a few viral particles are transmitted to a naïve host [30-32], investigations of the inter-host dynamics of equine influenza virus and norovirus have revealed inter-host transmission events to be characterized by a broad bottleneck [33,34]. NGS platforms have been used for investigations over time scales sufficient to incorporate the influence of intra-host scale immune pressures on RNA virus population diversity and subsequent transmission [30-34]. However, the insights that NGS technology can provide about the within and between host viral population dynamics of acute acting infections, particularly prior to the onset of a specific adaptive immune response, remain largely unexplored.

Utilizing Illumina NGS technology, this study investigates the evolutionary dynamics of FMDV intra*-* and inter*-*host transmissions during serial, acute infections (a “transmission chain”), both through time and across different samples from a host, prior to the onset of the adaptive immune response. Consensus level sequence changes in cattle have been previously defined using samples collected from an experimental transmission study [35], allowing transmission pathways to be reconstructed at the level of the individual animal. Due to the greater resolution offered by NGS, we were able to characterize the polymorphic structure of viral populations within samples collected from three hosts. These data were combined with those from a previous study of the initial inoculum material and first host [29], thereby constructing a chain of four individuals. We investigated the diversity and relatedness of virus within and between these host individuals, the dynamics of polymorphisms across the genome through time, and were able to compare the relative sizes of inter*-* and intra*-*host bottlenecks.

## Material and methods

### Transmission experiment and sample collection

**Figure 1:**
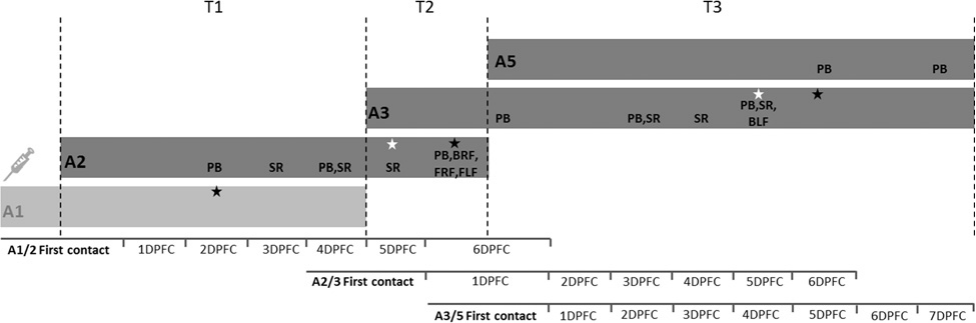
**Temporal scheme showing the contact transmission chain between the cattle in the experiment.** Figure highlights transmission between calves 1, 2, 3 and 5 (A1, A2, A3 and A5 respectively) with the three transmission events (T1 to T3) indicated. The time when the 18 samples from A2, 3 and 5) are shown (serum [SR]; probang [PB]; front left foot [FLF] lesion; front right foot [FRF] lesion; back right foot [BRF] lesion). One timeline for each transmission event is indicated, where days post first contact (DPFC) applies to the naïve calf in that transmission event. A five-pointed black star indicates when lesions appeared on all four feet and the equivalent white star indicates when the first foot lesions appeared.

The samples analysed were collected during an infection experiment where FMDV was passaged in series via direct contact through a group of four calves [35]. Calf 1 (A1) was inoculated intradermolingually with a dose of 10^5.7^ 50% tissue culture infective doses (TCID50) of FMDV (O_1_BFS 1860). The full-length FMDV genome sequence of this inoculum had previously been determined using Sanger sequencing (GenBank accession number EU448369). In addition, NGS data for selected samples originating from A1 have been previously described [29]. Twenty-four hours post needle-challenge, calf 1 (A1) was used to challenge naïve calf 2 (A2) by direct contact for a total of 4 days (transmission period 1 [T1] in the scheme in Figure 1). A1 was then removed from the experiment, and A2 was used to challenge naïve calf 3 (A3) by direct contact for 24 h (T2 in Figure 1). Following challenge, A2 was removed from the experiment. Successively*,* A3 was placed into direct contact with naïve calf 5 (A5) to be housed together for 14 days until study termination (T3 in Figure 1). Sequenced samples are indicated in Figure 1. Calf 4 (A4) was infected via indirect contact (35) and was not included in these analyses.

The sample types analysed included blood serum (SR), oesophageal-pharyngeal scraping (“probang”, PB) and foot-lesion epithelium samples, indicated as *XY*F, where *X* = {B,F} for Back and Front, and *Y* = {L,R} for Left and Right, and F for Foot. The nomenclature for these samples followed the notation A*n*-*m*DPFC-*Z*, where *n* = {2,3,5} represented the animal number in the chain, *m* was the number of days post first contact (DPFC) with an infected host for that particular animal, and *Z* was the sample type: for example, A2-4DPFC-SR corresponds to a serum sample taken from calf 2, 4 days after first contact with an infected host. Serum samples were taken daily and probang samples every other day. The consensus FMDV sequences for three of these samples (A2-2DPFC-PB, A2-4DPFC-PB and A2-6DPFC-PB) have been previously reported [35]. Foot lesion epithelium samples were collected within 24 h of first appearance. Daily rectal temperatures were monitored and clinical signs were defined here as any visible lesion or body temperature above 39.5°C.

### Genome amplification

**Figure 2:**
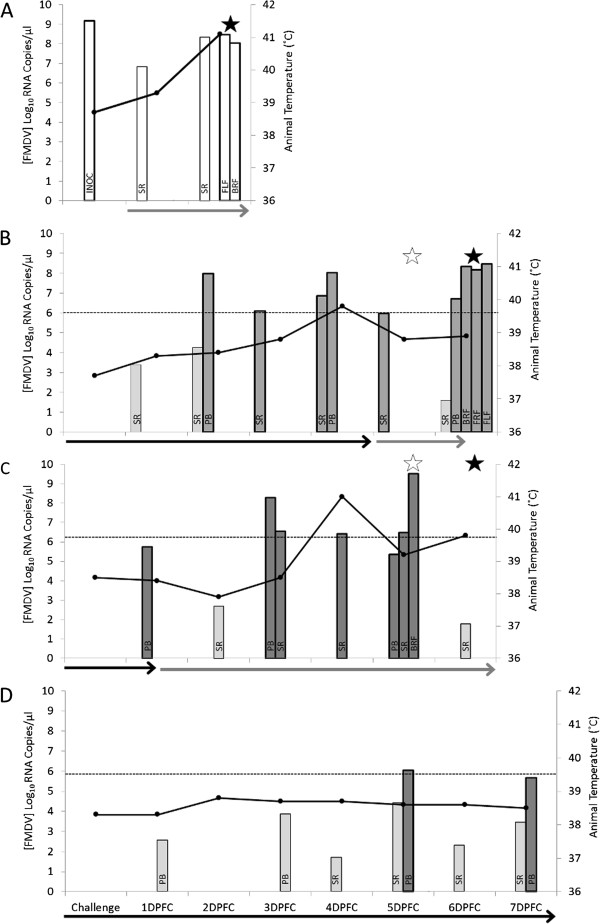
**Quantification of viral RNA copy number and clinical signs (temperature) of infected hosts.** FMDV RNA load in samples collected during the serial passage of FMDV through four calves, detected by real-time reverse-transcription polymerase chain reaction (rRT-PCR). Graph **A**–**D**, calf 1, 2, 3 and 5 (A1, A2, A3 and A5) respectively. A (A1) previously discussed in [29], sequenced samples in white with thick border and non-sequenced samples in white; **B**-**D** (A2, A3 and A5), sequenced samples in dark gray with thick border and non-sequenced samples in light gray. Inoculum (Inoc [A1 only]); serum (SR); probang (PB); front left foot (FLF) lesion; front right foot (FRF) lesion; back left foot (BLF) lesion; back right foot (BRF) lesion. Dashed lines indicate the minimum initial viral load to be amplified and then sequenced (10^6^ copies of FMDV RNA/μL of sample) for A2, A3 and A5. Gray arrows indicate the time the calf spent in contact with the next calf, while black arrows indicate the time spent in contact with the previous calf on the transmission chain. Animal temperatures are shown on the same graphs (black solid line). White stars indicates the day when the first foot lesions appeared (FRF and BLF for both A2 and A3), while black stars indicate the day at which lesions appeared on all four feet.

Total RNA was extracted (TRIzol, Invitrogen, Paisley, UK) from all biological samples collected from the experiment and quantified, as shown in Figure 2. Real-time reverse-transcription polymerase chain reaction (rRT-PCR) was performed to quantify FMDV genome copies in each of the samples, using an assay which can detect all serotypes of FMDV, as described previously [36]. rRT-PCR assays were performed on a Stratagene Mx3005P machine (Agilent Technologies, UK). For the generation of standard curves, a FMDV RNA standard was synthesized in vitro (MEGAScript T7, Ambion, UK) from a plasmid containing a 950 base pair insert of the 3D region of FMDV O/KUW/4/97 as described previously [37].

FMDV concentrations in each of the samples (A2, A3 and A5) were normalized to 10^6^ copies of FMDV RNA/μL prior to RT-PCR amplification for Illumina sequence analysis. Two genome fragments of FMDV were amplified using a protocol modified from that previously described [29]. Briefly, two independent reverse transcription reactions were performed for each sample. An enzyme with high fidelity (Superscript III reverse transcriptase, Invitrogen) was used in each reaction plus two FMDV specific primers (see Table 1) in order to reduce RT-introduced error and the risk of amplification bias. For each of these replicas, two PCR reactions generating long overlapping fragments (4065 bp and 4033 bp respectively) were carried out using a proof-reading enzyme mixture (Platinum Taq Hi-Fidelity, Invitrogen). For biosecurity reasons these individual fragments comprised <80% of the complete FMDV genome, and corresponded to nts 499–4563 and 4094–8126 of EU448369 (see Table 1 for PCR fragment and primer details). This enabled the amplified DNA to be transported outside of the high containment FMD laboratory for sequencing. The samples were amplified using the following cycling programme: 94°C (5 min), followed by 94°C (30 s), 60°C (30 s) and 72°C (4 min) for 39 cycles, with a final step of 72°C for 7 min. Where a sample fell within half a log below the 10^6^ copies of FMDV RNA/μL, neat (undiluted) sample was processed and sent for sequencing as long as it still yielded at least 700 ng of PCR product, samples below this threshold were not sequenced (as indicated in Figure 2).

**Table 1:** Oligonucleotide primers used for the amplification of the two overlapping FMDV genome fragments

	**Primer**^***a***^	**Primer sequence (5**^**′ **^**to 3**^**′**^**)**	**Location on genome**	**Amplicon size (bp)**
PCR set 1	OBFS-516+F	CCTTCGCTCGGAAGTAAAACGA	499-520^*b*^	4065
	OBFS 4563 R	CCCGCTGCTTTTCAAGGAT	4545-4563^*b*^	
PCR set 2	OBFS 4094 F	TCTCGACGAGGCCAAACC	4094-4111^*b*^	4033
	OBFS 8126 R	CTCCTAAGGTGTCGCGCG	8109-8126^*b*^	
RT 1	OBFS 8193 R	TTTTTTTTTTTTTTGATTAAGG	8172-8193^*c*^	-
RT2	OBFS 4926 R	AAGTCCTTGCCGTCAGGGT	4908-4926^*c*^	-

^*a*^ Last letter indicates a forward or reverse primer.

^*b*^ Numbering according to GenBank sequence EU448369.

^*c*^ Numbering according to GenBank sequence AY593815.

The fragments have the 5^′^ UTR S fragments omitted, up to and including the poly(C) tract, and overlap by 470 bp.

### Illumina sequencing

Independent replicate RT-PCR fragments for each sample were sequenced with the Genome Analyzer IIx (Illumina) maintained by Glasgow Polyomics facility at the University of Glasgow, according to the protocol as detailed in [29]. Following the temporal order in the transmission chain, the first 12 samples were multiplexed on the same lane, while the corresponding duplicate RT-PCR fragments were sequenced on a second lane, and ran on a different flow cell. The last 6 samples were multiplexed together on a lane belonging to a third flow cell. The 6 corresponding duplicates were multiplexed on a separate lane on the same flow cell.

### Filtering and alignment

Single-end reads were 70 nt long for the first 12 samples, and 73nt long for the last 6. Reads with unresolved nts or corrupted tags were removed from the analysis. We filtered the reads, removing any with an average probability of error per nt greater than 0.1% (probability of errors can be readily obtained from Illumina quality scores with the relation *p* = 1/(1 + 10^*Q*/10^), where Q is the quality score and *p* is the probability of error). We observed that the same strategy removed about 20% of the reads for the first 12 samples, but over 30% for the last 6 samples (see Additional file 1 for precise quantification). Moreover, we trimmed the reads to 65 nt for the first 12 samples, and to 70 nt for the last 6. The filtered, trimmed reads were aligned to FMDV genome O_1_BFS1860 (EU448369, the consensus sequence for the inoculum used to initiate the transmission chain) with a simple, custom-made scoring algorithm. No reads aligned ambiguously. For all subsequent analyses, we further trimmed the first and last 5 nts of each aligned reads, as they showed a higher number of mismatches to the reference sequence due to insertions or deletions close to the edges of the reads [29], and we masked all nts whose individual probability of error was higher than 10^-3^ (corresponding to quality scores of 30 or lower). Primer regions (detailed in Table 1) were also excluded from the analysis. Consensus sequences were always found to be identical between the two replicates for each sample. The genealogical relationships between consensus genomes were computed with the software package TCS [38] and reflected the most parsimonious genealogy. A schematic description of the steps in the analysis pipeline can be found in Additional file 2.

### Validation of low-frequency polymorphisms

The frequency of a polymorphism at a particular position in the genome in a viral population was defined as the frequency of mismatches in the aligned reads relative to the consensus of the inoculum (GenBank accession no. EU448369). A proportion of these mismatches were expected to be artifacts, arising from miscalled bases in the sequencing process. In order to distinguish between real and artifactual variation, we extended the validation method described in [29], summarized below. Under the assumption of independence, sequencing errors are binomially distributed, with the probability of observing *x*_*i*_ or more mismatches given by Binom(*x*_*i*_; *p*_*i*_/3, *n*_*i*_), where *x*_*i*_ is the number of nts bearing the most abundant mutation at site *i*, *n*_*i*_ is the coverage, *p*_*i*_ is the error probability computed from base qualities, and *p*_i_/3 represents the probability of the specific mutation observed in the reads. A score for site *i* was obtained, defined as s_*i*_=1-Binom(*x*_*i*_; *p*_*i*_/3, *n*_*i*_). We defined s_*i*,1_ to be the score obtained for the first replicate of the sample, and s_*i*,2_ the score obtained for the second replicate. Only sites where the most frequent mutation was the same in the two replicates, and where s_*i*,1_ < θ and s_*i*,2_ < θ, with θ being a threshold chosen to be >0.05, were validated and used for successive analyses. Finally, in order to minimize artefacts introduced through RT and PCR error, we considered only mutations at frequencies above 0.5% (choice based on the analysis of control data generated using an RNA clone, data not shown). The second most abundant mismatched nt exceeded 0.5% in both replicates at only 1 site across the 18 samples so we focus here only on the most abundant mismatches.

From each alignment we constructed the “mutation spectrum” which we define as a profile generated by the number of sites (y-axis) with a mismatch frequency of *x* (x suitably “binned” on the x-axis). This was viewed as a log-log plot.

### Genetic distance, entropy and dN/dS

Let *f*_*i,A*_ be the frequency of the most abundant polymorphism at position *i* in sample A, obtained as a weighted average of the two replicates {1,2}: *f*_*i*,*A*_ = (*f*_*i*,*A*,1_ * *n*_*i*,1_ + *f*_*i*,*A*,2_ * *n*_*i*,2_)/(*n*_*i*,1_ + *n*_1,2_), where *n*_*i*,1_ is the coverage of site *i* in the first replicate, and similarly for *n*_*i*,2_. Genetic distance between two samples A and B was computed with a population-wide measure $d=\sqrt{\frac{1}{N}\sum_{i=1}^{N}{\left(f_{i,A}-f_{i,B}\right)}^{2}}$, where *N* is the length of the sequence. Distances between samples were illustrated with a reduction to a two-dimensional space with classic (metric) multi-dimensional scaling, as implemented in the R software package; with this method, the distances between the points on the graph approximate the dissimilarities between the viral populations.

Similarly, the complexity of the viral populations was characterized by computing their Shannon entropy at each site, and then averaging over every site in the sequenced genome: for sample A, $S_{A}=\frac{1}{N}\sum_{i=1}^{N}\left[f_{i,A}\ln f_{i,A}+\left(1-f_{i,A}\right)\ln\left(1-f_{i,A}\right)\right]$. The genome-wide entropy measures the amount of “disorder” in the population, and it is maximum when all sites have perfectly balanced polymorphisms (i.e. *f*_*i,A*_=0.5 for all *i*).

In order to estimate the synonymous to non-synonymous ratio dN/dS, for each codon *i* in the ORF, we first computed the expected number of synonymous (*s*_*i*_) and non-synonymous (*n*_*i*_) sites. Then, for each read *j* covering entirely codon *i*, we counted the number of observed synonymous (*s*^*O*^_*ij*_) and non-synonymous (*n*^*O*^_*ij*_) substitutions with respect to the consensus sequence of the inoculum. Using all codons where *s*_*i*_>0 and $\sum_{j}s_{ij}^{o}>0,$we obtained an estimate for the number of synonymous substitutions per synonymous site, *p*_*S*_, and for the number of non-synonymous substitutions per non-synonymous site, *p*_*N*_, using the following equation: $p_{s}=\frac{1}{n_{\mathrm{cod}}}\sum_{i-1}^{n_{\mathrm{cod}}}\frac{1}{r_{i}}\sum_{j-1}^{r_{i}}\frac{s_{\ddot{\mathrm{\_U}}}^{o}}{s_{i}}$, where *n*_*cod*_ is the number of codons where the conditions above are met and *r*_*i*_ is the number of reads spanning entirely codon *i*. *p*_*N*_ was determined analogously. dN/dS was determined from *p*_*N*_ and *p*_*S*_ as described in [39].

## Results

### Quantification of viral titres

FMDV genome copies quantified by rRT-PCR of all the samples collected from the infected cattle (including the 18 samples analyzed in this study by NGS) are shown in Figure 2. During early stages of disease higher concentrations of viral RNA were measured in probang samples compared to serum samples. Viraemia*,* at 1–2 days post first contact, coincided with the clinical phase of disease. For A2 and A3 this correlated with the onset of fever and lasted up to 6 days after first contact with an infected host. As a consequence of being needle inoculated, the clinical phase of disease in A1 was shorter than that seen in subsequent animals. Conversely, the clinical phase of disease in A5 appeared elongated and less pronounced, as demonstrated by epithelial lesions not appearing on the feet until 8 and 9 days post first contact (not available for sequencing), as well as reduced fever and vireamia. The potential link between the elongated incubation period demonstrated in A5 and viral genetic mutations found within this animal is discussed further at the end of the next section.

Eighteen FMDV positive samples were sequenced from the sequential transmission chain in cattle: 9 from A2, 7 from A3 and 2 for A5. As the progenitor of this transmission chain, 2 samples from A1 plus the original inoculum (derived from a bovine tongue vesicle that had been extensively passaged in cell culture and used to artificially infect A1), previously described in [29], were also included in analyses and discussed where appropriate.

### Coverage and consensus genomes

Reads that passed the quality test were aligned to the consensus genome sequence of the original inoculum (FMDV strain O_1_BFS1860). The coverage of the different samples were influenced by the different multiplexing of the Illumina lanes, and ranged from 11605x (A2-4DPFC-PB, first replicate) to 32208x (A3-5DPFC-BLF, second replicate); precise figures can be found in Additional file 1. We computed the average frequency, for each mutation, that was weighted on the coverage received in the two replicates of each sample. Consensus-level mutations were defined as polymorphisms that appeared in more than 50% of this weighted average, with respect to the original inoculum by which the infection chain was initiated.

A total of 13 consensus-level mutations were present in the sequenced samples analyzed in this study, summarized in Table 2. Previous analysis of the samples collected from the inoculated calf A1 [29] identified one consensus-level mutation at position 2767, unobserved at this level in subsequent animals. Furthermore, two additional consensus-level mutations found in calf A1 in the 3^′^ UTR region (position 8134 and 8140) could not be followed in this study, as the modified RT-PCR fragments ended at position 8126 (omitting 36 nt of the 3^′^ UTR). Among the 13 mutations, one was present in every sample (site 2754, C->T). This mutation changes an amino acid residue in capsid protein VP3^56^ associated with heparan sulphate (HS) binding, as does position 2767 in A1 [29]. The inoculum used in this experiment had undergone extensive cell culture passage and, in common with other in-vitro adapted viruses, utilizes HS as a cellular receptor [40,41]. Subsequent replication in mammalian hosts drives the reversion of positively charged amino acid residues at specific sites in the viral capsid, which is then fixed in the host chain. Apart from this fixation event, two elements suggest the presence of neutral evolution (drift) in these samples since most consensus mutations appeared in only one sample (see Table 2), and the majority were synonymous (10/13) appearing at third codon positions (10/13). However, the impact of these individual mutations on viral fitness was not examined.

**Table 2:** Consensus-level mutations, and their characterization

**Position**	**Mutation**	**Frequency in sample**	**Gene**	**Syn/Nonsyn**^***a***^	**Ts/Tv**^***b***^	**Codon position**	**Sample**^***c***^
1087	C->T	54.4%	Leader	N: T->I	Ts	2	A2-2DPFC-PB
1164	A->G	63.9%	Leader	N: K->E	Ts	1	A2-6DPFC-PB
2417	C->A	51.1%	VP2	S: P->P	Tv	3	A2-6DPFC-PB
		52.8%					A3-3DPFC-PB
2754	C->T	> 60%	VP3	N: R->C	Ts	1	ALL BUT A1
2767	G->A	64.1%	VP3	N: G->D	Ts	2	A1-2DPFC-FLF
2768	C->T	52.8%	VP3	S: G->G	Ts	3	A3-3DPFC-PB
5435	C->T	> 55%	3A	S: G->G	Ts	3	ALL BUT A1 &
							A2-2DPFC-PB
							A2-3DPFC-SR
							A2-4DPFC-SR
							A2-5DPFC-SR
							A2-6DPFC-BRF
							A3-4DPFC-PB
5669	T->A	99.0%	3A	S: L->L	Ts	3	A2-6DPFC-FLF
5933	A->G	50.4%	3B2	S: K->K	Ts	3	A5-7DPFC-PB
6065	C->T	56.2%	3C	S: G->G	Ts	3	A3-1DPFC-PB
		99.7%					A3-3DPFC-SR
		99.3%					A3-4DPFC-SR
		75.6%					A3-5DPFC-PB
		99.6%					A3-5DPFC-SR
		99.7%					A3-5DPFC-BLF
		93.8%					A5-5DPFC-PB
		99.9%					A5-7DPFC-PB
6167	C->T	77.0%	3C	S: F->F	Ts	3	A2-6DPFC-FLF
7355	C->A	58.0%	3D	S: A->A	Tv	3	A2-2DPFC-PB
7376	T->C	54.4%	3D	S: D->D	Ts	3	A2-3DPFC-SR
		68.5%					A2-6DPFC-PB
		54.6%					A3-3DPFC-PB
7964	T->C	96.6%	3D	S: S->S	Ts	3	A3-3DPFC-SR
		97.6%					A3-4DPFC-SR
		53.4%					A3-5DPFC-PB
		99.1%					A3-5DPFC-SR
		99.9%					A3-5DPFC-BLF
		91.2%					A5-5DPFC-PB
		99.8%					A5-7DPFC-PB

^*a*^ Synonymous or Non-synonymous mutation with associated amino acid change.

^*b*^ Transition or Transversion.

^*c*^ Sample notation as described in the Material and Methods.

The mutation frequency is the weighted average frequency over the two sequencing runs for each sample.

When mutations were close enough on the genome to be spanned by a single read, we checked their co-occurrence (i.e. their presence on a single genome, or linkage). In the case of sites 2754 and 2768 in A3-3DPFC-PB, almost all the reads had independent nt substitutions compared to the reference genome. Moreover, two samples showing mutations at position 7376 (A2-3DPFC-SR and A2-6DPFC-PB) also exhibited a number of reads showing a mutation at position 7355 (~12% and 1% respectively), but almost no reads showed both sites mutated. We interpret this finding as demonstrating the co-circulation of two different variant genomes in the population, with two alternative mutations.

**Figure 3:**
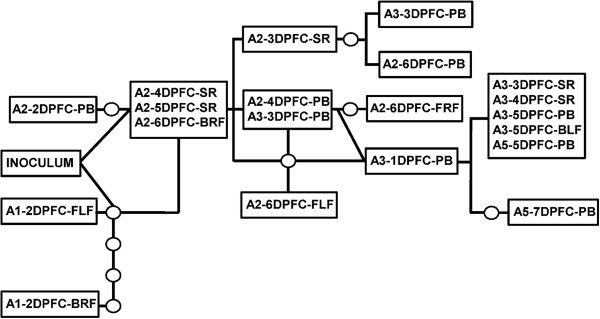
**Genetic network of the samples collected during the study.** Results shown are for consensus sequences using statistical parsimony implemented in TCS [38] using.

The relationships between the consensus sequences determined using statistical parsimony analysis (TCS [38]) are depicted in Figure 3. If mutations accumulated linearly during the infections, we would expect to see the viral consensus genomes to mirror the transmission chain, with clusters corresponding to different hosts. Instead, certain samples from different hosts shared the same consensus genome (a sample in A2 with a sample in A3; late samples in A3 with a sample in A5). Moreover, intra-host samples varied substantially and gave rise to dead-end branches of the networks, corresponding to mutations that did not transmit further down the chain.

Finally, we saw no evidence at the consensus level of mutations within the non-structural genes that would suggest attenuation of the virus, as previously demonstrated during serial passage of FMDV in pigs [12], to explain the observed elongated incubation period in calf A5. Although impacts on genome secondary structure cannot be ruled out with such data, due to lack of polymorphism linkage, this elongated incubation is more likely a result of reduced infective dose, indirectly indicated by the reduced viral RNA copy number measured within samples from this host. However, incubation period is highly variable for FMDV and is dependent on a number of factors in addition to infective dose including route of transmission, therefore the precise cause of this variation is not clear in this instance. All animals investigated tested negative for antibodies against FMDV serotype O by both the Ceditest (Cedi Diagnostics B. V.) and solid phase competition ELISA [42], thereby ruling out the influence of an adaptive humoral immune response by these animals.

### Sub-consensus mutations

Having demonstrated that populations in different samples in a host can differ at the consensus level, we extended our analysis to minority variants at each genomic site, using the high coverage obtained with deep sequencing.

**Figure 4:**
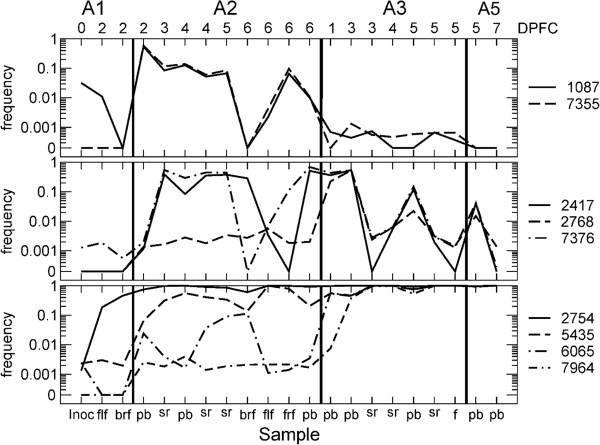
**Changes in frequency for mutations reaching consensus level in the experiment.** Data shown is for 9 representative sites (out of 13 in total) where at least one sample in the experiment reached the level of the consensus. Results are divided according to patterns. Top panel: Mutations present in A2 and then gradually lost in the next hosts. Middle panel: Mutations prevalently present in probang samples and sera, across all hosts. Bottom panel: Mutations reaching fixation.

First, we looked for the presence of the 13 consensus-level mutations in all samples (A2, A3 and A5), to determine whether they were present as minority variants. We found that this was the case as shown in Figure 4 for nine of these mutations grouped by their differing dynamics. These patterns are compatible with a neutral model, where the frequencies of mutations vary in time and the states at 0 and 100% frequency are absorbing. The dynamics of the four additional consensus-level mutations are displayed in Additional file 3, together with the single consensus-level mutation previously found in host A1 at site 2767. Additional file 4 depicts the frequencies of the polymorphisms across the genome, for all the samples.

**Figure 5:**
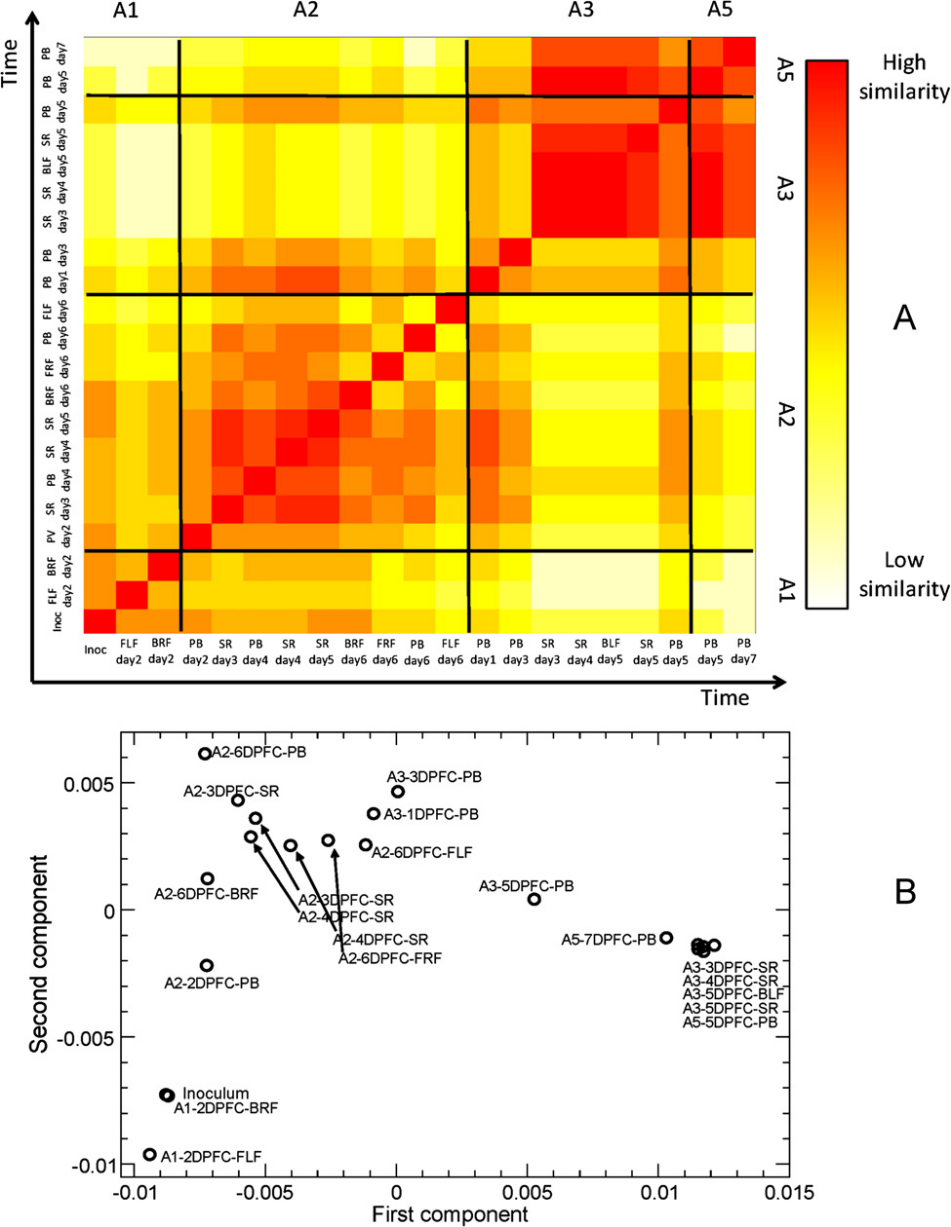
**Genetic heterogeneity revealed by deep-sequence analysis.** Panel **A**: Distances between viral populations collected in hosts A1, A2, A3 and A5, obtained considering all validated mutations at frequencies above 0.5%. A2 presents a large heterogeneity, with the FLF samples being very different from all others. Conversely, A3 shows remarkably similar pattern to late samples, while the early probangs bear a larger similarity with the A2 samples. Samples in A5 are very similar to several late A3 samples. Panel **B**: Metric two-dimensional multidimensional scaling analysis of the distance matrix: the data formed the characteristic horseshoe pattern, sign of a latent order in the data.

Viral populations can be more closely related than their consensus sequence suggests. Using the polymorphic frequencies at each site we estimated the genetic distance between different viral populations (Figure 5A). Boundaries between hosts did not always correspond to a sudden increase in the distance measures. In particular, early samples of A3 are more related to samples in A2 than to later samples in the same host. Late samples in A3, in turn, are very similar to samples in A5. Finally, samples like A2-6DPFC-FLF are very different from everything else, suggesting an evolutionary trajectory in this population which did not propagate through the infection chain.

The minimum distance between A3 and samples of A2 collected at 6DPFC is found between samples A2-6DPFC-FRF and A3-1DPFC-PB: based solely on this observation we would conclude that the viral population transmitted to A3 derived from the A2 FRF lesion. However, a closer inspection of the time series shows that the minimum distance between hosts A2 and A3 is found between A2-5DPFC-SR and A3-1DPFC-PB. Moreover, sample A3-1DPFC-PB has a comparable low distance from samples A2-4DPFC-SR, A2-4DPFC-PB and A2-3DPFC-SR. Finally, the presence of a consensus level mutation at site 6167 in A2-6DPFC-FRF, which was not found at any significant frequency in any A3 samples analysed here, reduces the probability that the transmitted viral population was seeded directly from this foot lesion. Considering all these observations, a likely scenario is that infection occurred around day 5 through a viral population originating from the upper oesophagus and pharynx of A2, thus through airborne spread. Around the same time, other subpopulations originating in the oesophageal-pharyngeal region seeded the feet lesions, where the virus underwent independent replication and diverged from the sample passed on to A3. Moving on to the infection from A3 to A5, the situation is less clear: A5-5DPFC-PB was close to a number of A3 samples, including two serum samples, the back right foot lesion and, to a lesser extent, a late probang (the absolute minimum found with A3-3DPFC-SR). As samples are very similar to each other, resolution is limited and we cannot disprove either a direct infection route originating from a foot lesion in A3 or an infection originating from a population similar to that found in the probang.

An easier visualization of the distance relationships between samples is obtained with a standard metric multi-dimensional analysis in two dimensions (displayed in Figure 5B). From this, for example, it is clear that the infection of A5 could have originated from any of the late samples in A3. The observed “horseshoe” pattern is typical of dimensionality reduction techniques, and is the sign of a latent ordering of the data, namely the accumulation of mutations along the transmission chain [43].

### Inter- and intra-host bottlenecks

If a bottleneck is narrow, only a few viral particles found a new population. Consequently, mutations included in the founding population will be likely fixed in the new population. A population founded as a result of a narrow bottleneck could then be recognized by a depletion of sites with intermediate polymorphic frequencies in the mutation spectrum. Conversely, in the case of a wide bottleneck, the diversity of the founding population is a good representation of the diversity of the ancestral population, and we should then expect to see the mutations at intermediate frequencies well preserved in the new population. This criterion can be used to qualitatively assess the size of the founding population in each of our samples. Here, we considered both intra-host bottlenecks (i.e. events leading to the founding of a new lesion in a distant epithelium) and inter-host bottlenecks (i.e. events leading to a host-to-host transmission).

**Figure 6:**
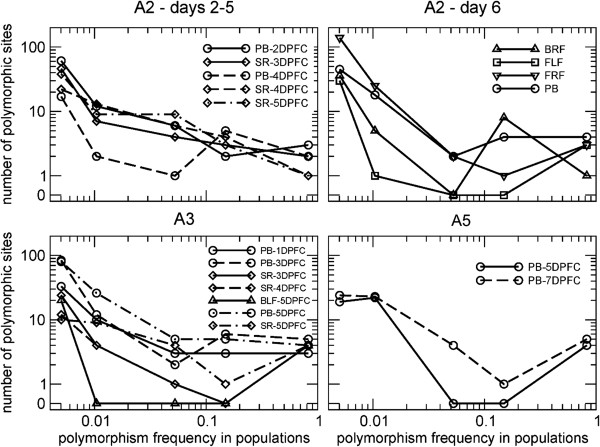
**Mutation spectra of samples collected from the cattle.** Plots (for each animal) represent the abundance of mutations at frequencies above 0.5% across the different samples: in some cases (typically probangs and sera) the mutation spectrum smoothly decreases in abundance as the frequency of mutations increases. However, in some samples (typically feet), the intermediate frequency region is depleted, suggesting narrow bottlenecks.

Figure 6 displays the mutation spectra, defined as the collection of mutated sites, segregated into individual bins according to their frequencies, for all samples in calves A2-A5. In all the feet lesions of A2 and A3 a characteristic spectrum was observed that had a depletion of mutations at intermediate frequencies. This observation is consistent with the hypothesis that these populations underwent a narrow intra-host bottleneck. We speculate that this pattern originates from the combination of low-frequency mutations created in recent rounds of replication and mutations at consensus level, present in the founding population, and fixed by genetic drift. On the other hand, A3-1DPFC-PB, the earliest sample in A3, representing a population that has recently passed through a host-to-host bottleneck, does not show this depletion, suggesting that the transmission to A3 arose as a result of the transfer of a sizable viral population from A2: however, alternative explanations cannot be ruled out, such as the occurrence of multiple transmission events. A probang sample taken from A5 at 5 days post first contact was the earliest sample from this animal that contained the minimum initial viral load of 10^6^ copies of FMDV RNA/μL. A5-5DPFC-PB shows again the typical pattern corresponding to narrow bottlenecks. Surprisingly, the viral population had not recovered its complexity sufficiently to include a full range of mutation frequencies at 5 days post first contact; however, the prolonged incubation period observed in A5, together with the observation that the calf showed no vireamia until 4DPFC, support again the hypothesis of transmission to A5 through a narrow bottleneck. We therefore speculate that in our infection chain, intra-host bottlenecks were narrower than host-to-host bottlenecks.

### Entropy and dN/dS

**Figure 7:**
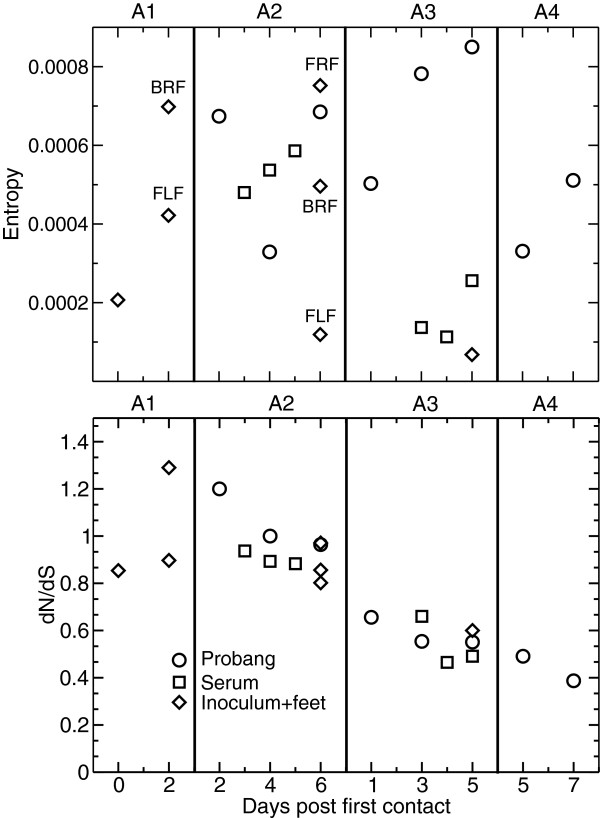
**Shannon entropy (left panel) and dn/ds (right panel), across all samples.** Validated mutations at frequencies above 0.5% were included in these analyses. The complexity of viral populations fluctuates across samples, with lower values often found in correspondence of foot lesions. dn/ds ratios show a clear decreasing trend along the transmission chain.

The complexity, or diversity, of a viral population can be measured using the Shannon entropy of a sample of genomes. Diversity can be acquired in two ways: 1) through the presence of many low frequency polymorphic sites across the genome, where a single nucleotide is largely dominant, and 2) through fewer but more balanced polymorphic sites where multiple nucleotides are more equitably represented. Samples founded by a small initial population typically have not recovered from the loss of complexity associated with a narrow bottleneck and so should have low entropy (although exceptionally high-levels of replication could lead to high entropy through route 1). Conversely, samples founded by a large seeding population should display higher entropy, as they retain most of the diversity of the original population. Figure 7a shows entropy for all the samples. The values fluctuate considerably: the lowest values are observed in the feet (host A2 and A3), reinforcing the hypothesis that these are “young” populations that have experienced a narrow bottleneck. However, the entropy of foot lesion A2-6DPFC-FRF is high: this value is reached through the very large number of polymorphic sites at frequencies around 0.5% found for this sample (see Figure 6, note the log scale on the y axis) suggesting that this lesion was founded by a slightly larger population, and that early replication introduced numerous new mutations at low frequencies.

Early probang samples in A3 and the first probang in A5 available for sequencing show intermediate values of entropy. For A3, where the probang sample was taken only 1 day post first contact, the value observed, together with the absence of depletion in the mutation spectrum discussed above, supports the hypothesis that this complexity was inherited from an ancestral population through a wide bottleneck.

Finally, we evaluated the evolutionary dynamics of FMDV through the chain by computing the non-synonymous to synonymous ratio (dN/dS) for all the samples in this study (see Figure 7b). We found a monotonic reduction in dN/dS through the transmission chain, across all the samples collected from all tissues. While the values of dN/dS were close to 1 in A2, suggesting a dominant role for random genetic drift, it steadily decreases in A3 and A5, where the viral populations appear to undergo a continuous purifying selective pressure.

## Discussion

Samples from a sequential infection experiment were analyzed using Illumina technology. The samples were collected at different time points during the infection of each host. While foot lesions comprised a relatively spatially-discrete source of virus, probangs (oesophageal-pharyngeal scrapings) are thought to be composed of several infection foci (as well as those infected earliest), including the oesphagus, pharynx and oral cavity, and therefore are often more heterogeneous than samples taken from feet lesions. Going beyond the resolution afforded by Sanger sequencing methods, the Illumina technology allowed us to investigate the fine details of the polymorphic viral samples collected. In particular, we were able to use this information to compare the size of intra- and inter-host transmission bottlenecks and to determine the most likely lesion that passed on the infection.

Consensus sequencing is a valuable tool that can be used to reconstruct the sequential accumulation of nt substitutions between hosts and provide evidence for the transmission of virus across an epidemic outbreak. However, consensus sequencing has limited resolution to differentiate between samples collected at the intra and inter-host scale: we observe identical consensus sequences within the same host (A2, 3 samples and A3, 4 samples) and between hosts (A2 and A3; A3 and A5). We used deep sequencing to monitor low-frequency variation at specific sites in early samples prior to their appearance as consensus-level substitutions in later samples. This approach revealed patterns of mutations which drifted over and under the consensus threshold (50% of the reads) through time. This observation, together with the dynamics of the 13 consensus-level mutations generated during the transmission chain (four reached fixation, two were lost and seven appeared only in some samples), suggests close to neutral selection pressures and a dominant role for random genetic drift. We examined the linkage between mutations that could appear on the same read, and demonstrated that several viral genotypes can co-circulate in a lesion, as suggested by previous work [44]. These data suggest that every host harbors multiple populations evolving in time and differing at one or more sites, and those samples obtained from different hosts are not necessarily representative of what is transmitted.

Investigation of the mutation spectra provided evidence for variation in the polymorphic structure of viral populations. In particular, we speculate that there are two types of founding events: intra-host, when the infection reaches a distant epithelium through the blood stream, and inter-host, when the infection is transmitted to the next host. In this experiment, several related lines of evidence point toward narrow bottlenecks during the process of virus dissemination during intra-host infections and a wider bottleneck for the inter-host transmissions. These include: 1) distances between viral populations which were sometimes larger within hosts compared to between hosts, suggesting that the size of founding populations within a host may be relatively small; the small distance between some populations in sequentially infected hosts is consistent with host-to-host transmission events seeded by large viral populations, where representative samples of the diversity in the ancestor population is passed on to the next host; 2) the mutation spectra of populations sampled early during the infection of a host exhibited polymorphisms across a range of frequencies, while those of newly-formed lesions at the end of the clinical phase displayed a depletion of polymorphisms with intermediate frequencies; and 3) the Shannon entropy of populations did not drop substantially across hosts but was often low in samples recovered from “younger” foot lesions.

Analysis conducted with mutation spectra, at the host-to-host scale, also showed a strong trend in dN/dS towards an increased purifying selective pressure along the chain. If a role for the adaptive immune response is ruled out, we can hypothesize that the declining dN/dS ratio results from the elimination of mildly deleterious mutations generated early in the chain. We conclude that host-to-host transmissions can be seeded by populations of different sizes, while in all cases examined, seeding of a distant host epithelium lesion occurred via a small founding population. Numerous in vitro studies have demonstrated loss of FMDV fitness with cell-culture passage due to the accumulation of deleterious mutations [45-47], an observation that was mirrored during serial passage of FMDV in pigs [12]. However, reduced vireamia, such as that observed in A5 and as discussed during the serial passage of FMDV in sheep [48], may be explained by alternative mechanisms other than bottlenecking, including isolate-specific infection dynamics and viable transmission rates.

In the present study, we considered only polymorphisms at frequencies higher than 0.5%. The coverage obtained by NGS allowed us to investigate lower frequencies, but at the likely price of introducing significant numbers of artifactual mutations into the analysis. Accordingly, we note that Shannon entropy was computed in [29] for A1 samples in a slightly different manner: to avoid contamination by low-frequency artifactual mutations, we considered here only the contribution deriving from the dominant polymorphism at each site. The entropy of the original inoculum, computed according to the method used in this work then becomes 2.07 × 10^-4^, while we obtain 4.22 × 10^-4^ and 6.98 × 10^-4^ for the A1 FLF and BRF lesions, respectively. These values are compatible with those found later in the transmission chain, confirming that a single host passage results in a cell-cultured population acquiring complexity (as measured by the Shannon entropy) equivalent to a natural in vivo infection. While polymorphisms at frequencies below 0.5% are unlikely to change the conclusions of the present study, a more comprehensive understanding of the population genetics of acute RNA virus infections will require quantifying polymorphic frequencies well below this threshold. Such understanding will require either direct high fidelity sequencing of RNA without amplification, or more detailed study and reduction of the errors introduced by the RT-PCR process and sequencing reactions themselves.

Taking multiple samples from the different hosts allowed us to see a host as a collection of potential sources of infection rather than harboring a single heterogeneous population. The different populations, while clearly related showed different levels of heterogeneity, potentially caused either by tissue/organ-specific amplification or bottlenecking and founder effects during intra-host viral spread. While the ability to recognize a single lesion as a source of infection is limited to the samples available and by the extent of mixing between populations via the blood stream, characterizing multiple potential source populations is a clear advancement. This information could be a powerful tool to reconstruct more refined transmission trees and develop a more sophisticated understanding of how viral genetic differences accumulate with transmission events.

## Competing interests

The authors declare that they have no competing interests.

## Author’s contributions

MJM generated methods to analyse the sequence data and undertook analyses of the sequence data. CFW undertook experimental work to optimise and process samples for sequence analysis. MJM and CFW wrote the draft manuscript. NJK participated in the analysis of the sequence data. NJ and DJP designed and undertook experimental infection study that provided the cattle samples used in this study. DPK and DTH conceived and designed this study. All authors were involved in the preparation and review of the final manuscript.

## Supplementary Material

Additional file 1: Table S1 Details and metrics for Illumina data. Table outlines the number of raw and filtered reads and coverage for both replicates of each sample. Click here for file

Additional file 2: Table S2 Analytical pipeline: Brief descriptions of the steps in the pipeline used to analyse next-generation sequencing data. Click here for file

Additional file 3: Figure S1 Frequencies across samples for 4 samples. Frequencies across samples of the four remaining mutations reaching consensus in one sample only (for the nine mutations described in the main text, see Figure 4), together with site 2767, previously found mutated in the inoculated calf A1. Top panel: Mutations prevalently present in the probangs. Bottom panel: Mutations present at high frequency in a single sample (6167 is present in a second sample at about 10% frequency). Click here for file

Additional file 4: Figure S2 Frequencies of mutations across the genome. Results were computed with respect to the initial inoculum. Click here for file
