# Supplementary material for: Evolution of foot-and-mouth disease virus intra-sample sequence diversity during serial transmission in bovine hosts
Source: Vet Res. 2013 Mar 1;44(1):12. doi: 10.1186/1297-9716-44-12 (PMC3630017; doi:10.1186/1297-9716-44-12)
Supplement: Additional file 1: Table S1 — Details and metrics for Illumina data. Table outlines the number of raw and filtered reads and coverage for both replicates of each sample. [file 1297-9716-44-12-S1.doc]

| Sample | #read (1) | #filtered read (1) | Coverage (1) | #read (2) | # filtered read (2) | Coverage (2) |
| --- | --- | --- | --- | --- | --- | --- |
| A2-2DPFC-PB | 2790963 | 2128716 | 15351x | 2101069 | 1709078 | 12325x |
| A2-3DPFC-SR | 2807388 | 2168346 | 15637x | 2626534 | 2162173 | 15592x |
| A2-4DPFC-PB | 2092694 | 1609323 | 11605x | 2797204 | 2311235 | 16667x |
| A2-4DPFC-SR | 2720760 | 2076448 | 14974x | 2073010 | 1722013 | 12418x |
| A2-5DPFC-SR | 3336753 | 2542656 | 18336x | 2974976 | 2463627 | 17766x |
| A2-6DPFC-BRF | 2561453 | 1962495 | 14152x | 2650213 | 2214366 | 15967x |
| A2-6DPFC-FLF | 2704138 | 2085893 | 15042x | 2657830 | 2207046 | 15916x |
| A2-6DPFC-FRF | 2550724 | 1958710 | 14125x | 2626899 | 2188872 | 15785x |
| A2-6DPFC-PB | 2249190 | 1691592 | 12199x | 2607965 | 2142547 | 15451x |
| A3-1DPFC-PB | 2752115 | 2140025 | 15432x | 2326107 | 1930781 | 13924x |
| A3-3DPFC-PB | 2458092 | 1870211 | 13487x | 2059365 | 1705933 | 12302x |
| A3-3DPFC-SR | 2691979 | 2075898 | 16330x | 2926522 | 2411961 | 18974x |
| A3-4DPFC-SR | 4746119 | 2761230 | 21721x | 5450750 | 3778399 | 29723x |
| A3-5DPFC-BLF | 5311265 | 3079516 | 24225x | 6000979 | 4094216 | 32208x |
| A3-5DPFC-PB | 4353393 | 2469838 | 19429x | 5598961 | 3724627 | 29300x |
| A3-5DPFC-SR | 5231498 | 3049485 | 23989x | 5611303 | 3891223 | 30611x |
| A5-5DPFC-PB | 5444899 | 3238943 | 25479x | 5622686 | 3931106 | 30924x |
| A5-7DPFC-PB | 5420473 | 3249013 | 25559x | 4858806 | 3410646 | 26830x |

**Table S1 Details on illumina data: number of raw and filtered reads and coverage for both replicates of each sample**.
