# Supplementary material for: Evolution of foot-and-mouth disease virus intra-sample sequence diversity during serial transmission in bovine hosts
Source: Vet Res. 2013 Mar 1;44(1):12. doi: 10.1186/1297-9716-44-12 (PMC3630017; doi:10.1186/1297-9716-44-12)
Supplement: Additional file 2: Table S2 — Analytical pipeline: Brief descriptions of the steps in the pipeline used to analyse next-generation sequencing data. [file 1297-9716-44-12-S2.doc]

**Table S2** Brief descriptions of the steps in the pipeline used to analyse next-generation sequencing data.

| Stage 1 | Raw Reads | (demultiplexed) |
| --- | --- | --- |
| Stage 2 | Filtering | Removing all reads with average quality score < 30 (corresponding to probability of error of 0.1%) |
| Stage 3 | Trimming | Removing last nucleotides of each read (3-5 according to quality scores) |
| Stage 4 | Alignment | Aligning the reads to the O1BFS1860 FMDV genome with a simple custom-made scoring routine. Reads with 5 or more mismatches were discarded. |
| Stage 5 | Trimming | Trimming the first and last 5 nucleotides of the aligned reads to remove indels |
| Stage 6 | Masking | Removing from analysis every nucleotide with quality score < 30 (corresponding to probability of error of 0.1%) |
| Stage 7 | Consensus genomes | Determination of consensus genomes by counting the most abundant nt in the reads at every genomic position |
| Stage 8 | Validation | Statistical validation of observed polymorphisms, based on a binomial null distribution. Polymorphisms at frequencies <0.5% were discarded because potentially due to amplification artefacts |
| Stage 9 | Analysis | Generation of quantity of interest: mutation spectra, population distances, Shannon entropy, dN/dS |
