# Supplementary figures and images for: Evolution of foot-and-mouth disease virus intra-sample sequence diversity during serial transmission in bovine hosts
Source: Vet Res. 2013 Mar 1;44(1):12. doi: 10.1186/1297-9716-44-12 (PMC3630017; doi:10.1186/1297-9716-44-12)

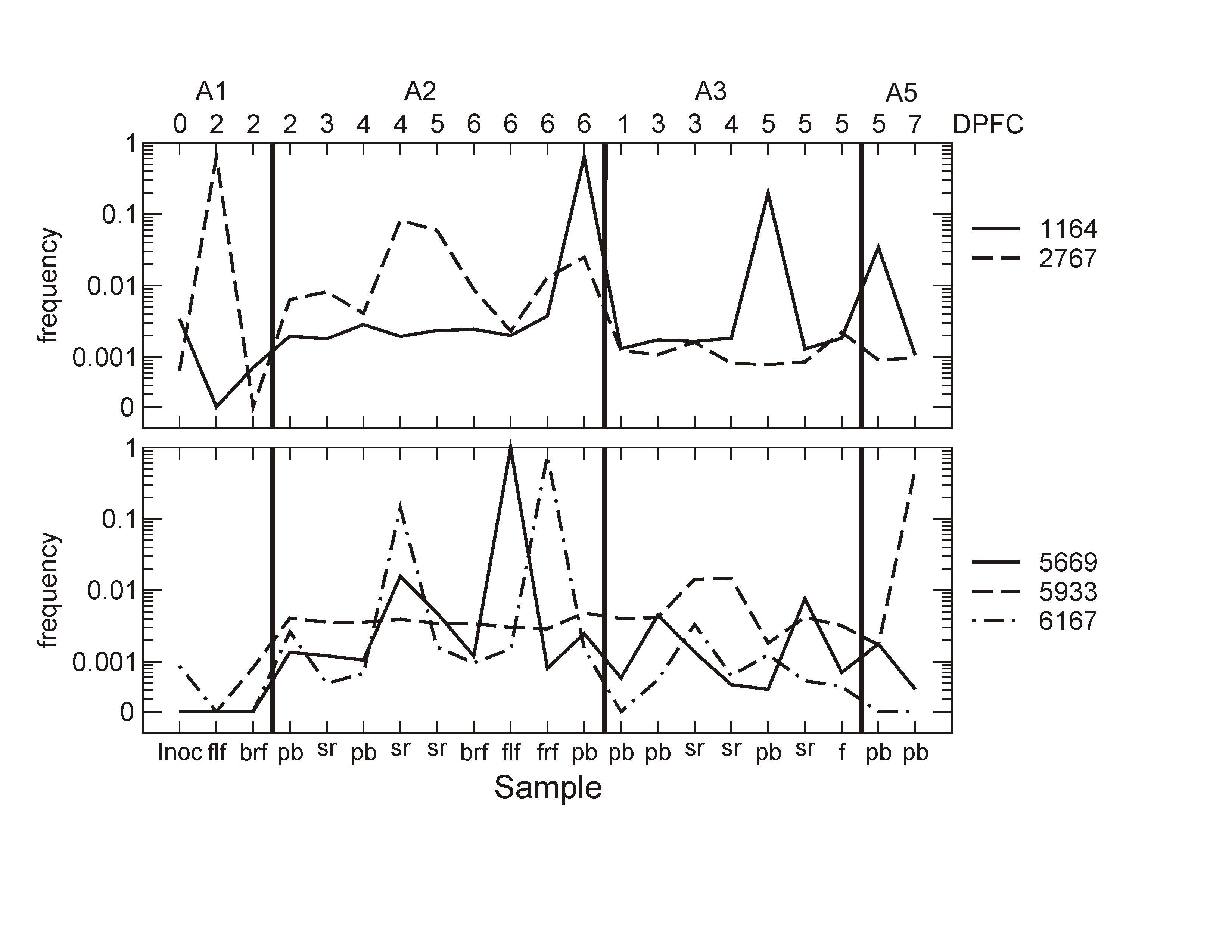

Supplement: Additional file 3: Figure S1 — Frequencies across samples for 4 samples. Frequencies across samples of the four remaining mutations reaching consensus in one sample only (for the nine mutations described in the main text, see Figure 4), together with site 2767, previously found mutated in the inoculated calf A1. Top panel: Mutations prevalently present in the probangs. Bottom panel: Mutations present at high frequency in a single sample (6167 is present in a second sample at about 10% frequency). [file 1297-9716-44-12-S3.doc]

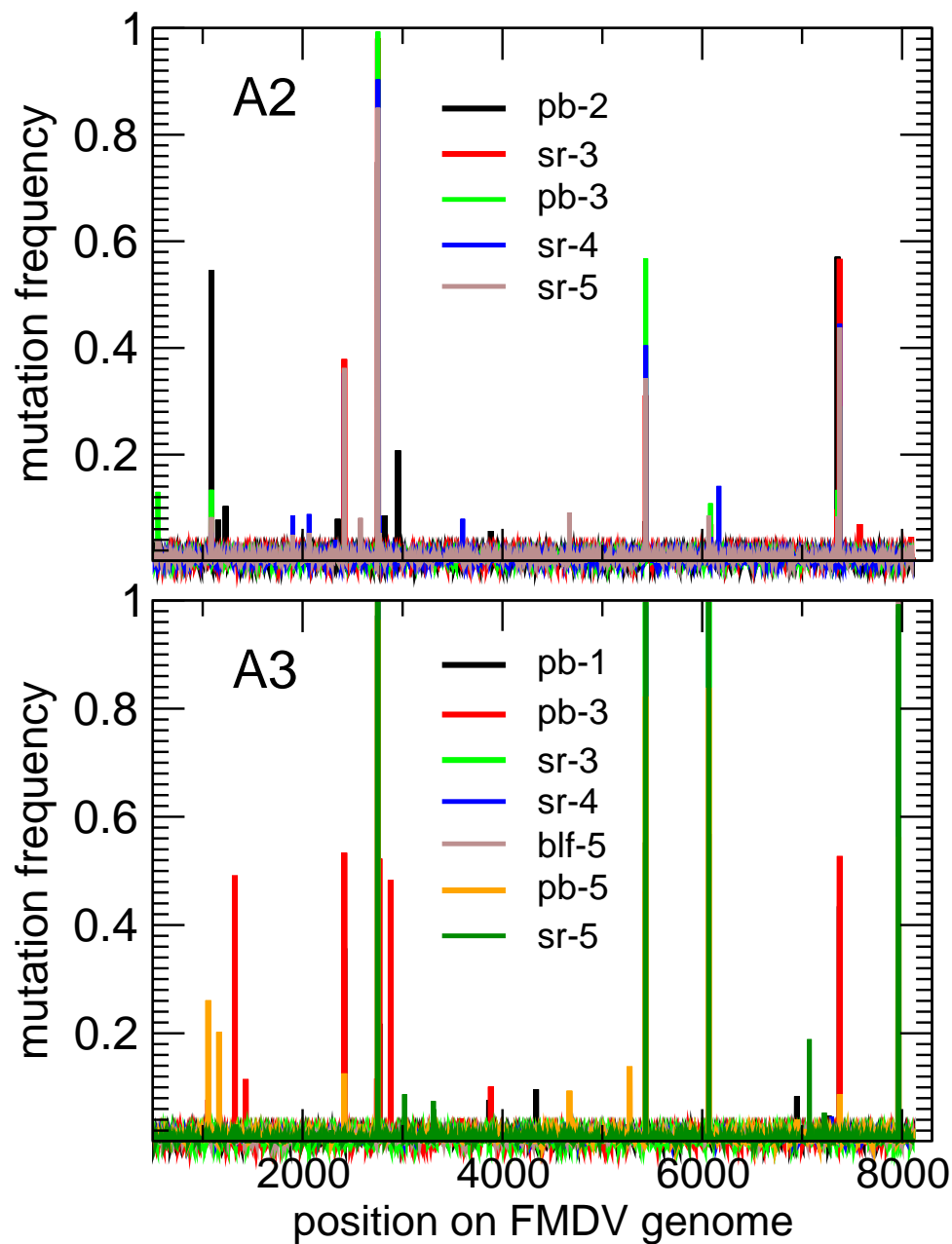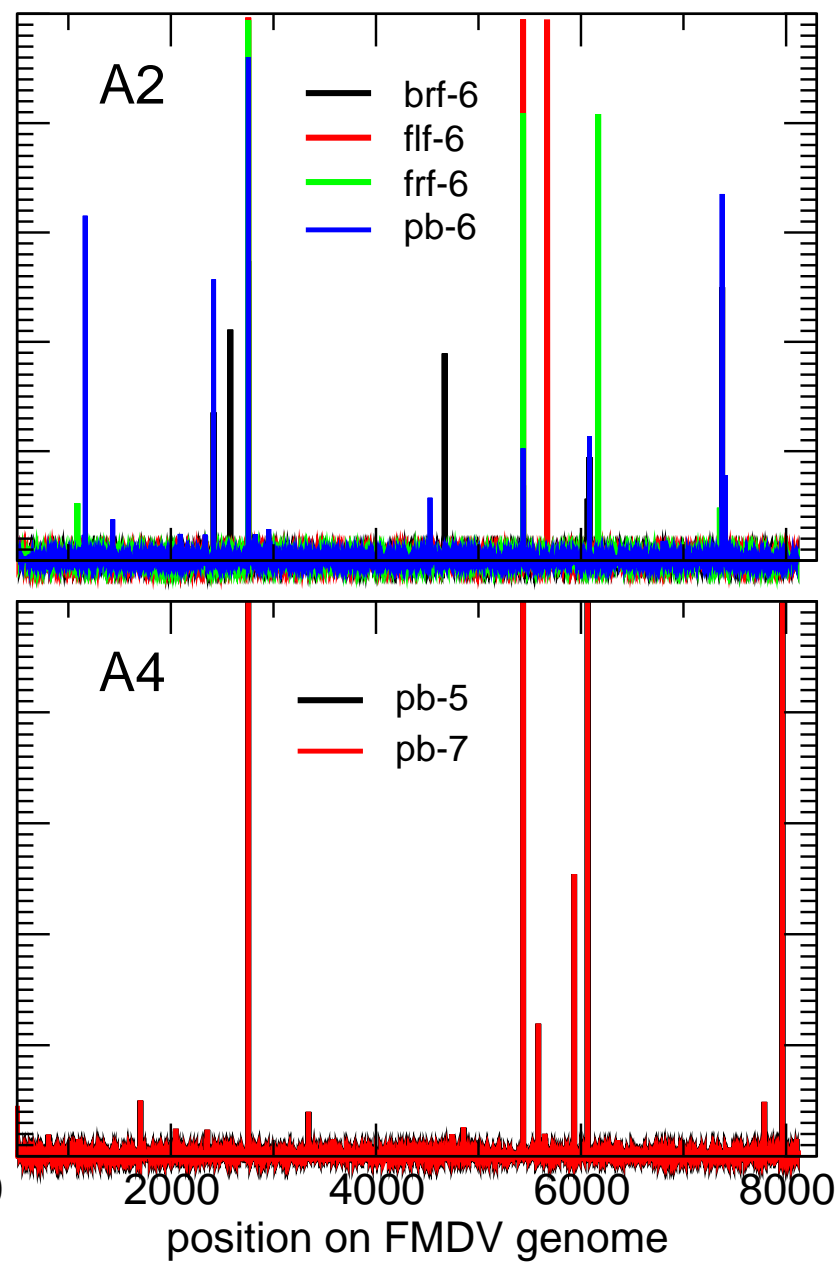

Supplement: Additional file 4: Figure S2 — Frequencies of mutations across the genome. Results were computed with respect to the initial inoculum. [file 1297-9716-44-12-S4.pdf]
